# Predicting the Outcome of Voriconazole Individualized Medication Using Integrated Pharmacokinetic/Pharmacodynamic Model

**DOI:** 10.3389/fphar.2021.711187

**Published:** 2021-10-13

**Authors:** Ping Yang, Wei Liu, Jiajia Zheng, Yuanyuan Zhang, Li Yang, Na He, Suodi Zhai

**Affiliations:** ^1^ Department of Pharmacy, Peking University Third Hospital, Beijing, China; ^2^ Department of Laboratory Medicine, Peking University Third Hospital, Beijing, China

**Keywords:** voriconazole, individual pharmacokinetic parameters, population pharmacokinetic, Monte Carlo simulation, minimal inhibitory concentration (MIC)

## Abstract

Therapeutic drug monitoring is considered to be an effective tool for the individualized use of voriconazole. However, drug concentration measurement alone doesn’t take into account the susceptibility of the infecting microorganisms to the drug. Linking pharmacodynamic data with the pharmacokinetic profile of individuals is expected to be an effective method to predict the probability of a certain therapeutic outcome. The objective of this study was to individualize voriconazole regimens by integrating individual pharmacokinetic parameters and pathogen susceptibility data through Monte Carlo simulations The individual pharmacokinetic parameters of 35 hospitalized patients who received voriconazole were calculated based on a validated population pharmacokinetic model. The area under the concentration-time curve for free drug/minimal inhibitory concentration (fAUC_ss_/MIC) > 25 was selected as the pharmacokinetic/pharmacodynamic (PK/PD) parameter predicting the efficacy of voriconazole. The cumulative fraction of response (CFR) of the target value was assessed. To verify this conclusion, a logistic regression analysis was used to explore the relationship between actual clinical efficiency and the CFR value. For the 35 patients, the area under the free drug concentration-time curve (fAUC_ss_) was calculated to be 34.90 ± 21.67 mgh/L. According to the dualistic logistic regression analysis, the minimal inhibitory concentration (MIC) value of different kinds of fungi had a great influence on the effectiveness of clinical treatment. It also showed that the actual clinical efficacy and the CFR value of fAUC_ss_/MIC had a high degree of consistency. The results suggest that it is feasible to individualize voriconazole dosing and predict clinical outcomes through the integration of data on pharmacokinetics and antifungal susceptibility.

## Introduction

Voriconazole is a broad-spectrum antifungal agent commonly used to treat invasive aspergillosis and candidiasis infections (Allegra et al., 2018; Ikeya et al., 2018). The most commonly used dose for adults in our country is 4 mg/kg or 200 mg twice daily (Chen et al., 2018). Because of the nonlinear pharmacokinetic behavior of voriconazole, in clinical practice, about 15–28% of patients failed to respond to treatment even if administered in the same manner, and about 12.5% of patients had severe adverse reactions (Taylor et al., 2003). Voriconazole showed significant individual differences in the plasma concentrations according to the recommended dosage regimens (Wang et al., 2014). Based on the complex pharmacokinetic behavior and the narrow therapeutic window of voriconazole (Xu et al., 2016a), it is necessary to strengthen the therapeutic drug monitoring (TDM) and individualized medication management.

Therapeutic drug monitoring is considered to be an effective tool for the individualized use of voriconazole (Dolton et al., 2012; Huurneman et al., 2016; Perreault et al., 2018). However, relying only on the information of Pharmacokinetics (PK) to guide individual drug administration had great limitations, because it doesn’t take into account the susceptibility of the infecting microorganisms to the drug. Therefore, this study tried to establish the optimal voriconazole dosage regimen both considering the PK and pharmacodynamics (PD) factors (such as the minimal inhibitory concentration (MIC) distribution) and predicting the clinical outcomes.Monte Carlo simulation (MCS) is a calculation method based on the “random number” (Schmidt et al., 2008). It can simulate the real system according to the appropriate model, and obtain information that would be difficult to acquire through experimentation. The MCS is widely used for PK/PD parameter evaluation and dosing regimen optimization. It could predict the probability of target attainment (PTA) or the cumulative fraction of response (CFR) for pathogen response to different dosing regimens, which would be useful for the determination or optimization of an effective clinical dosing regimen (Li et al., 2016). In the present study, Monte Carlo simulation was performed to determine the probability of reaching a target AUC_ss_/MIC ratio of voriconazole. The dosing regimens were evaluated and determined by estimating the CFR. It has been demonstrated that the area under the concentration-time curve for free drugs (fAUC_0→24h_)/MIC >25 could be used as an indicator for the efficacy evaluation of voriconazole (Andes et al., 2003; Liao et al., 2015).

In this study, the MCS was conducted to evaluate the dosing strategies based on the target fAUC/MIC of voriconazole against *Aspergillus spp.* and *candida* in patients of Peking University Third Hospital who were treated with voriconazole. Previous MCSs studies have typically used population pharmacokinetic (PPK) parameters (e.g., popfAUC) to calculate and predict the target value (Xu et al., 2016a; Box et al., 2018; Liu et al., 2019; Ren et al., 2019), which might be appropriate for a region or certain types of people. So far, however, there has been little discussion about probability simulation of treatment outcome using individual’s PK/PD parameters. This is the first study achieving requisite PK/PD target by MCS using individual pharmacokinetic parameters. The simulation results are accurate, targeted and suitable for the formulation of individualized drug regimen. By integrating of data on pharmacokinetics and antifungal susceptibility, this approach can guide clinicians in selecting appropriate antibiotic regimens.

## Materials and Methods

### Research Objects and Data Sources

Patients receiving intravenous or oral voriconazole for the prevention or treatment of invasive fungal infection during hospitalization in hematology department from 2018 to 2020 were enrolled in the study. The exclusion criteria were as follows: 1) age < 18 years, 2) pregnancy, 3) patients with abnormal liver and kidney function. The patient’s laboratory examination data and CYP2C19 genotyping were obtained from our electronic medical record system of Peking University Third Hospital. Demographic data, administration of voriconazole, invasive fungal infections (IFIs), clinical outcomes, were also recorded.

### Blood Sampling and Analytical Assay

The C_trough_ blood samples (30 min predose) were collected after steady-state from each patient. The concentration of voriconazole in plasma samples was determined by a sensitive and specific liquid chromatography-tandem mass spectrometry (LC-MS/MS) method validated previously in our laboratory (Zhang et al., 2020). Calibration standard responses were linear over the range of 50–10,000 ng/mL. A lower limit of quantification was 50 ng/ml, and the intra-run and inter-run precisions were both within 4.4%.

### Pharmacokinetics

Numerous population pharmacokinetic studies on voriconazole have been conducted recently.(Shi et al., 2019). Voriconazole is extensively metabolized by and is also an inhibitor of the cytochrome P450 (CYP) isozymes (Friberg et al., 2012), and the polymorphism of CYP2C19 is one of the factor for the pharmacokinetic changes. By screening the published population pharmacokinetic studies of voriconazole, four candidate literatures were included (Hope, 2011; Liu and Mould, 2014; Wang et al., 2014; Lin et al., 2018), and finally the model suitable for Chinese patients and considering CYP2C19 polymorphism was selected for the calculation of personalized pharmacokinetic parameters in this study (Wang et al., 2014). The PPK model could be described as follows: CL = 6.95×[1–0.012×(AGE-61)]×(1–0.37×PM)×[1–0.0016×(ALP-104)]×e^ƞ1^, V = 200×[1 + 0.0098×(AGE-61)]×e^ƞ2^, Ka = 1.1. Where, ƞ is the relationship between the individual and the population typical value, which is normally distributed with mean 0 and variance ω^2^. In the literature, ω_cL_ = 0.287, ω_V_ = 0.254 (Wang et al., 2014).

Model performance was evaluated by creating goodness-of-fit (GOF) plots, including individual predictions (IPRED) and population predictions (PRED) versus the observed values and plots for conditional weighted residuals (CWRES) versus PRED, and CWRES versus time after dose. Meanwhile, the visual predictive check (VPC) method was also used for external validation of the model. For the VPC, the data set was simulated 1,000 times using the population Pharmacokinetic model parameters (Zhao et al., 2013). The simulated concentrations (5th, 50th and 95th percentiles) and the corresponding observed concentrations were described against time.

### Calculation of Individual Pharmacokinetic Parameters of Patients

The population pharmacokinetic model was carried out in the pharmacokinetic software NONMEM (version 7.2.2, ICON Development Solutions, Ellicott City, MD, United States ), the PK parameters (clearance, CL) of 35 patients were estimated using the population pharmacokinetic model and bayesian estimate. The protein binding of voriconazole was set as 58% (Bellmann and Smuszkiewicz, 2017) for the calculation of fAUC values. The fAUC_ss_ of voriconazole in patients was calculated using the formula (fAUC_ss_ = F ×DOSE × *f*/CL). Where, DOSE is daily DOSE (mg/d); F is bioavailability, in this study, F value was set to 1, f was set to 0.58 (Liu et al., 2019), and CL is the clearance.

### The MIC Distribution of Voriconazole to *Aspergillus Fumigatus* and *Candida*

The MIC distribution of voriconazole to *aspergillus fumigatus* was obtained from literature (Luo et al., 2017).The MIC distribution of *candida* was obtained from the clinical laboratory department of our hospital, which was shown in Table 1. The percentage of MIC distributions was used for each simulation to calculate the cumulative fraction of response (CFR).

**TABLE 1:** The frequency distribution of MIC values of voriconazole against *Aspergillus fumigatus* and *Candida albicans* (%).

Microbial species	Number of isolated strains	MIC										
		0.015	0.03	0.06	0.125	0.25	0.5	1	2	4	8	16
*aspergillus fumigatus*	2,778	0	0.04	0.58	4.43	42.94	39.27	10.48	1.4	0.61	0.25	0
*Candida*	290	0	0	77.93	9.31	7.59	8.17	0	0	0	0	0

### The Monte Carlo Simulation

The area under the concentration–time curve and the MIC (fAUC_ss_/MIC) was chosen as the PK/PD index for voriconazole therapeutic effectiveness. The Oracle Crystal Ball^®^ (V.11.1.2.2) was used to perform Monte Carlo simulation for each patient with pharmacokinetic parameters and the MIC distributions. The MCSs were iterated 5,000 times to estimate the CFR for pharmacodynamic exposure (Lim et al., 2018) (fAU_ss_/MIC) using weighted summation, which was used to support dose selection decisions. During simulations, MICs followed a discrete distribution. The fAUC followed a lognormal distribution. The variability came from the random effect parameter of the applied PPK model. The CFR value > 90% was considered optimal for a dosage regimen to reach a target of fAUC_ss_/MIC>25. The CFR value ≥ 90% was defined as predicted clinically effective, while the CFR value < 90% was defined as predicted clinically ineffective.

### Statistic Methods and Determination of Clinical Outcome

The relationship between predictive probability of clinical efficacy and C_trough_, relationship between predicted CFR value and clinical efficacy were analyzed by logistic regression implemented with SPSS software (version17.0). The Odds Ratios (OR) were obtained using binary logistic regression model. For treatment of invasive fungal infections (IFI), a successful clinical outcome was determined to be a complete or partial response to the clinical signs and symptoms of invasive fungal infection, or a serologic outcome and negative fungal culture results. The clinical outcome of failure was 14 days after voriconazole treatment, stable IFD (symptoms and signs of the disease, no improvement in survival, stable radiosurgery, negative serology), progressive IFD (worse symptoms and signs of the disease, worsening radiosurgery), and even death. For IFI prevention, the absence of breakthrough fungal infection was considered a successful clinical outcome, otherwise was considered an outcome of failure.

## Results

### Demographic Characteristics of Patients and Voriconazole Plasma Concentration Data

In total, 35 patients were incorporated in the study. Among them, 16 patients were treated with voriconazole for the invasive fungal infections (IFI), and the remaining 19 were treated to prevent infection. All of the 35 patients were treated with voriconazole orally. The observed plasma concentration was from 0.03 to 7.94 mg L^−1^. Demographic and clinical data were listed in Table 2.

**TABLE 2:** demographic parameters for 35 patients and voriconazole plasma concentration data.

Parameter	Value/mean ± SD (range)
Voriconazole C_trough,ss_ (mg·L ^−1^)	2.15 ± 1.74 (0.03–7.94)
Gender (male/female)	24/11
CYP2C19 phenotype EM:IM:PM	16:15:4
Patient age/years	47.80 ± 17.42 (19–91)
ALP/U·L ^−1^	113.23 ± 53.56 (40–274)
Indication for therapy (n (%) of patients)	
Antifungal prophylaxis	19 (54.29%)
Empirical therapy	16 (45.71%)

C_trough,ss_: the trough concentrations at steady-state. EM:extensive metabolize, IM: intermediate metabolizer, PM:poor metabolizer; For CYP2C19 *2 *3 *17 phenotyping, *2/*2, *2/*3, *3/*3:PM; *1/*2, *1/*3, *2/*17,*3/*17:IM; *1/*1:EM; ALP: Alkaline phosphatase.

### Population Pharmacokinetic Model Suitability Evaluation

GOF plots were used to evaluate the fitness of the model (Supplementary Figure S1). The IPRED agreed with the dependent values (DV) well. The CWRES in the model were uniformly distributed within the accepted range (y = ± 2).

The VPCs were based on 1,000 simulations on the retrospective data for the PPK model (Supplementary Figure S2). The purple shaded areas in this figure indicate the 90% confidence interval of the 5th and 95th of the simulated data, the pink shaded areas show the 90% confidence interval of the 50th of the simulated data. The observed data and the simulated data have similar distribution characteristics. The VPC plots associated with the selected model indicated a good agreement between the observed and predicted data.

### Individual Pharmacokinetic Parameters

The individual pharmacokinetic parameters of 35 patients were estimated by the population pharmacokinetic model. CL = 9.16 ± 6.51 L/h, fAUC_ss_ = 34.90 ± 21.67 mgh/L.

### Monte Carlo Simulation

**FIGURE 1:**
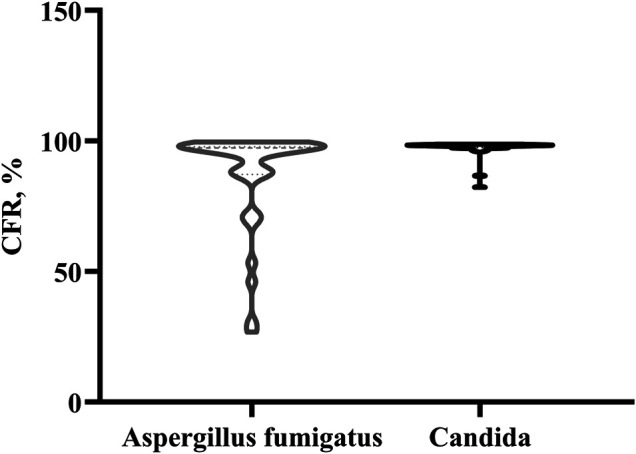
The violin plot of CFR distribution for *Aspergillus fumigatus* and *Candida.*

Monte Carlo simulation was performed using a PK/PD target of fAUC_ss_/MIC>25. The CFR values distribution in the case of existing empirical dosage regimen, for *Aspergillus fumigatus* and *Candida* were displayed as a violin plot (Figure 1). It shows a widely dispersed values of the CFR distribution for *Aspergillus fumigatus*, while for the case of *Candida*, the CFR values were concentrated between 82.4–98.9%.

### Logistic Regression Analysis

The predicted clinical effectiveness for prevention and treatment of *aspergillus fumigatus and candida* infection was calculated. The logistic regression analysis showed a relationship between the predicted clinical outcome and C_trough_, described as Eq. 1 and Eq. 2. (OR 14.99; 95% CI 2.4–93.2; *p* = 0.004 and OR 5.3E58; 95% CI 0-; *p* = 0.982, respectively).

$$Ln\frac{P}{p-1}=-3.751+2.707C_{trough}$$ (1)

Where, *p* was the probability of predicted clinical effectiveness for prevention and treatment of *aspergillus fumigatus* infection.

$$Ln\frac{P}{p-1}=-20.462+135.21C_{trough}$$ (2)

**FIGURE 2:**
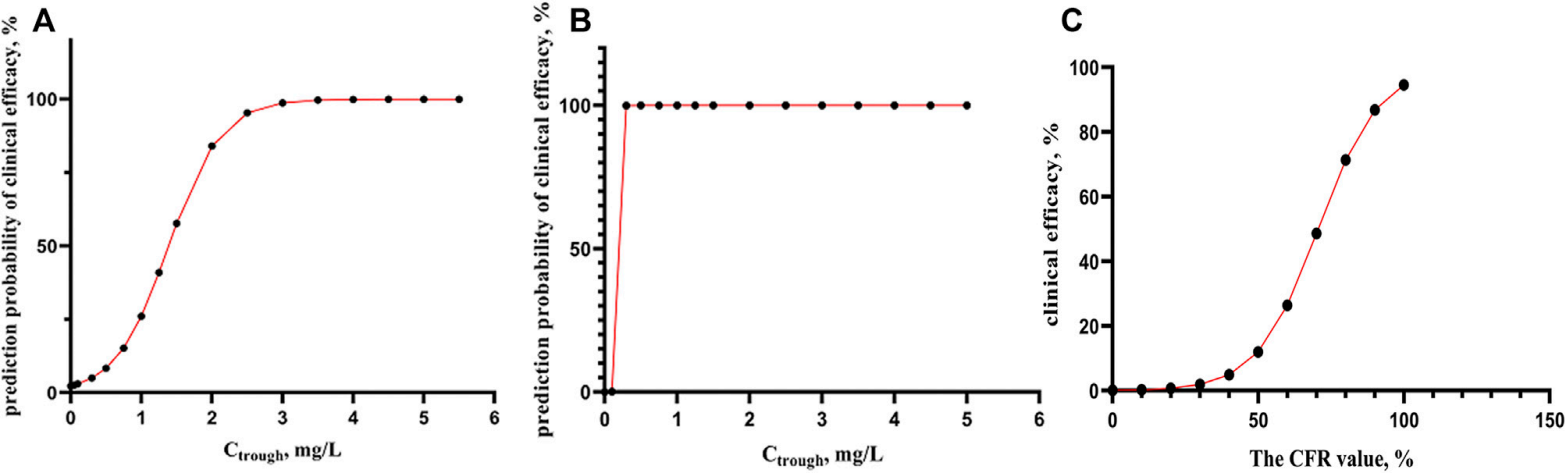
**(A-B)** the correlation between predict probability of clinical efficacy and C_though_, for prevention and treatment of *aspergillus fumigatus and candida* infection using logistic regression analyses (*n* = 35). **(C)** The correlation between the actual clinical efficacy and CFR value of fAUC_ss_/MIC using logistic regression analyses (*n* = 16).

Where, *p* was the probability of predicted clinical effectiveness for prevention and treatment of *candida* infection.The correlation between the probability of predicted clinical effectiveness and C_though_, for prevention and treatment of *aspergillus fumigatus and candida* infection using logistic regression analyses were shown in Figures 2A,B.

As from Figure 2A, for prevention and treatment of *aspergillus fumigatus*, when C_trough_ > 2 mg/L, the probability of predicted clinical effective was able to reach more than 85%, while in the case of *candida* (Figure 2B), At very low C_trough_ value, 99% of probability of clinical effective can be obtained.

Among the 35 patients, 16 patients receiving voriconazole were for the treatment of IFD, and the actual clinical effectiveness was observed. The logistic regression analysis showed the correlation between actual clinical outcomes and the CFR value of fAUC_ss_/MIC >25, the OR was 1.10 (95% CI 1.0–1.2; *p* = 0.036).

$$Ln\frac{P}{p-1}=-6.847+0.097CFR\%$$ (3)

Where, *p* was the probability of actual clinical effectiveness.

For Eq. 3, there was a strong correlation between CFR values and actual clinical outcomes (*p* = 0.036), and the accuracy of predicting the effective clinical treatment is 90.9%, and ineffective clinical treatment is 60%. It can be seen from Figure 2C that when CFR> is 90%, the probability of actual clinical effectiveness is also close to 90%.

## Discussion

Therapeutic drug monitoring (TDM) of voriconazole concentrations plays an important role in maximize efficacy and to minimize adverse events(Miyakis et al., 2010). However, during the treatment of deep fungal infection with voriconazole, the efficacy of the drug was suboptimal in some patients even though the plasma concentrations of voriconazole appeared to be within the therapeutic window, implicating that the plasma concentration alone is insufficient in decision making. Therefore, it is very important to consider PK, PD (microbiological parameters) in individualized medication. In this research, a population pharmacokinetic model was used to calculate the individual pharmacokinetic parameters of patients, then MCSs is applied to forecast the probability of clinical efficacy. Based on therapeutic drug monitoring, considering the pharmacokinetic characteristics of individual patients, and MIC values for specific fungi to predict the likelihood of a treatment result can improve the clinical effectiveness of voriconazole. The importance and originality of this study are that it explores a new application of Monte Carlo simulation in individualized drug administration strategy.

The result of the dualistic logistic regression analysis showed the relation between the predicted clinical effectiveness of fAUC_ss_/MIC and C_trough_ for prevention and treatment of *aspergillus fumigatus and candida* infection. For Eq. 1, the 95% Cl in Eq. 1 is relatively large. This might be related to the small sample size. The statistical accuracy for predicting the two outcomes was 92.3 and 95.5%, respectively (*p* < 0.05), suggesting that Eq. 1 is appropriate to describe the relationship between C_trough_ and probability of predicted clinical effectiveness in the case of *aspergillus fumigatus* infection. For Eq. 2, the 95% CI could not be estimated. The reason might well be that the number of predicted clinical failure groups was too small (2 cases) to meet the statistical requirements. Eq. 2 may be not appropriate for predicting the probability of clinical outcome in the case of *candida* a infection (*p* = 0.982) in this study. However, we can still see the overall trend between C_trough_ and the probability of the predicted treatment effectiveness. It can be seen that the MIC value has a great influence on the effectiveness of clinical treatment.

The relationship between the real clinical efficiency and the CFR values of fAUC_ss_/MIC>25 was also built based on the logistic regression model in this study. The result showed that there was a statistically significant and positive correlation between the two variables. This result was a verification of the proposed prediction methods in our study. The complete chain of evidence for PK/PD studies from “drug concentration - PK/PD target - clinical outcome” was achieved. At the same time, we also tried to use C_trough_ as covariate for the observed clinical efficacy in logistic regression. The result showed that the correlation between C_trough_ and actual clinical outcomes was not significant (*p* = 0.994). This result was also compatible with the point of view that we could not predict clinical outcomes based on patient’s C_trough_ alone. Therefore, in the clinical treatment and prevention with voriconazole, we should consider both C_trough_ and the CFR value of fAUC_ss_/MIC>25. It’s worth noting that when the C_trough_ was between 0.5 and 2 μg/ml, we recommend obtaining the accurate MIC for the patient, and calculating the AUC/MIC value. If AUC/MIC<25, the dose could be increased. If AUC/MIC≥25, the current dose is maintained. In general, high blood drug concentrations are associated with high CFR values. The situation of C_trough_ greater than 2 while CFR less than 90% are unlikely to occur.

It is suggested that the MIC value of voriconazole against the fungus for each patient should better be detected at the initial stage of treatment. If the MIC value for each person is available, we can calculate the AUC_ss_/MIC directly without the process of Monte Carlo simulation. But in reality, that might be difficult to realize. The proposed approach based on CFR would help the dose guidance if MIC data for the causative pathogen is not provided. In this study, the MIC distribution of *candida* was narrow and the geometric mean was small. Therefore, in the MCSs simulation, most of the CFR values were above 90%, to reach the goal value of fAUC_ss_/MIC >25. Therefore, for the case with very small MIC, the significance of MCSs simulation may be less than the concentration monitoring results.

An accurate and predictive PK/PD model is an extremely powerful tool to find the needed dose to meet efficacy breakpoints (Schmidt et al., 2008). In Chen’s study, logistic regression model showed a high correlation between voriconazole C_min_/MIC ratio and clinical response (Chen et al., 2016). In Mangal’s research (Mangal et al., 2018) both preclinical (fAUC_24_/MIC≥25) and clinical (C_trough_/MIC >2) PK/PD index of efficacy yielded similar probability of target attainment. In the existing studies based on PK/PD models, the predicted dose of voriconazole against aspergillus was different with that against *Candida* albicans (Wang et al., 2014; Xu et al., 2016b; Chen et al., 2016; Mangal et al., 2018).

There were a couple of limitations in this study: the sample size was insufficient, and this may lead to no statistically significant difference in the logistic regression analysis. Besides, the enrolled patients were all from the department of hematology, which might have introduced some bias in the result.

Taken together, this study has proposed a new PK/PD approach that would further improve the antifungal management. However, there might be situations that patients develop serious side effects of voriconazole or their symptoms are not improved despite having drug levels with the therapeutic concentration range or achieving the optimal fAUC/MIC ratio. In this regard, a further study is needed to explore the possibility of integrating the PK/PD data with clinical indicators, which may include the improvement of clinical signs and symptoms, or occurrence of adverse reactions.

## Conclusion

The individual PK/PD parameters and therapeutic drug monitoring are equally important in the individualized administration of voriconazole for patients. The results suggest that it is feasible to individualize voriconazole dosing and predict clinical outcomes through the integration of data on pharmacokinetics and antifungal susceptibility.

## Data Availability

The raw data supporting the conclusion of this article will be made available by the authors, without undue reservation, to any qualified researcher.

## Supplementary Material

Supplementary Figure S1 The goodness-of-fit (GOF) plots of the PPK model: individual predictions (IPRED) and population predictions (PRED) versus the observed values and plots for conditional weighted residuals (CWRES) versus PRED, and CWRES versus time after dose.

Supplementary Figure S2 Visual predictive checks for the PPK model. Solid lines indicate the 50th percentile of the observed data, dash lines indicate the 5th and 95th percentile of the observed data. The purple shaded areas in this figure indicate the 90% confidence interval of the 5th and 95th of the simulated data, the pink shaded areas show the 90% confidence interval of the 50th of the simulated data from the PPK models.
