# Supplementary material for: Predicting the Outcome of Voriconazole Individualized Medication Using Integrated Pharmacokinetic/Pharmacodynamic Model
Source: Front Pharmacol. 2021 Oct 13;12:711187. doi: 10.3389/fphar.2021.711187 (PMC8548711; doi:10.3389/fphar.2021.711187)
Supplement: Supplementary file 1 [file Table1.DOCX]

Table S1. Summary of Population pharmacokinetic models in adult for voriconazole.

| NO. | Population mean | Inter-individual variability | Residual variability | Reference |
| --- | --- | --- | --- | --- |
| 1 | CL(L·h^-1^)=6.95×[1-0.012×(AGE-61)]×(1-0.37×PM)×[1-0.0016×(ALP-104)]×e^η1^  V(L)= 200×[1+0.0098×(AGE-61)]×e^η2^  K_a_(h^-1^) = 1.1 | 0.287  0.254 | proportional (%):10.8;  additive (mg·L^-1^)  :0.016 | Wang et al., 2014 |
| 2 | K_m_(µg/ml)=1.15  V_max,1_ (g/h/70 kg^e^)=113×(WT/70)^0.75^  Logit(*V*_max,inh_)=1.50  T_50_=2.42  CL (L/h/70 kg)=5.3×(WT/70)^0.75^  V_2_(L/70 kg)=77.6×(WT/70)  V_3_(L/70 kg)=89.5×(WT/70)  Q(L/h/70 kg)=15.9×(WT/70)^0.75^  Logit(F_1_)=0.595  Ka(h^-1^)=1.2  A_lag_=1  Rate (mg/h)=12.8 | 1.91  1.91  NS  0.634  0.139  0.831  0.459  0.713  NS  0.910 | σ_IV_^2^:0.53  σ_oral_^2^:0.61 | Liu and Mould, 2014 |
| 3 | Ka(h^-1^) = 9.77  V_max,1_(mg·h^-1^)=37.67  K_m_(mg·L^-1^)=2.07  Vol(L)=149.11  Kcp(h^-1^)=2.01  Kpc(h^-1^)=9.35  F=0.86  Lag(h)=1.12 | SD  10.3  11.19  1.11  173.49  3.33  11.68  0.13  0.99 |  | Hope, 2011 |
| 4 | CL(L·h^-1^)=2.88×exp(PM=0)×exp[0.45×(IM=1)]×exp[0.80×(EM=1)]×exp(η_CL_);  V(L)=169.27×(WT/56.1)^1.30×exp(η_V_);  F(%)=0.58×exp(POT_1_=0)×exp[0.43× (POT_2_=1)]×exp[0.57×(POT_3_=1)]×exp[0.57×(POT_4_=1)] × exp(η_F_) | 0.42  0.39  0.22 | σ:0.57 | Lin et al., 2018 |

NO.1: CL, clearance; V, volume of distribution; AGE, age; WT:weight; ALP, alkaline phosphatase;K_a_:the absorption rate constant;

NO2: K_m_, Michaelis-Menten constant; V_max;1_, maximum elimination rate at 1 h after start of dosing; T_50_, time (in hours) at which half of the maximum inhibition occurs; CL, linear clearance; V_2_, central volume of distribution; V_3_, peripheral volume of distribution; Q, inter-compartmental clearance; K_a_, first order absorption rate constant; A_lag_, absorption lag time; NS, not supported in the model;

NO.3: K_a_, the first-order rate constant that connects the gut with the central compartment; V_max_, the maximum rate of enzyme activity of voriconazole, K_m_, the concentration of voriconazole where clearance is half maximal; K_cp_ and K_pc_, the first-order intercompartmental rate constants connecting the central and peripheral

Compartments; F, the bioavailability, and Lag is the absorption time.

NO.4: CL, clearance; V, volume of distribution; WT, weight; EM, extensive metabolizer; IM, intermediate metabolizer; PM, poormetabolizer; POT, postoperative time.
