# Supplementary figures and images for: Predicting the Outcome of Voriconazole Individualized Medication Using Integrated Pharmacokinetic/Pharmacodynamic Model
Source: Front Pharmacol. 2021 Oct 13;12:711187. doi: 10.3389/fphar.2021.711187 (PMC8548711; doi:10.3389/fphar.2021.711187)

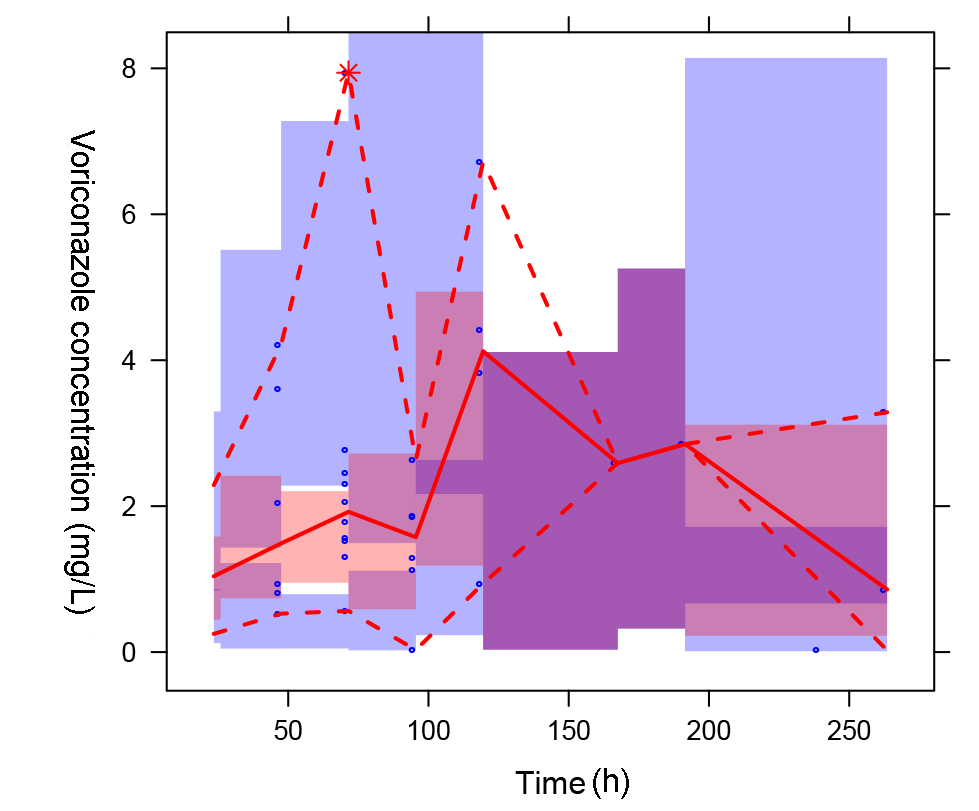

Supplement: Supplementary file 2 [file Image2.TIF]

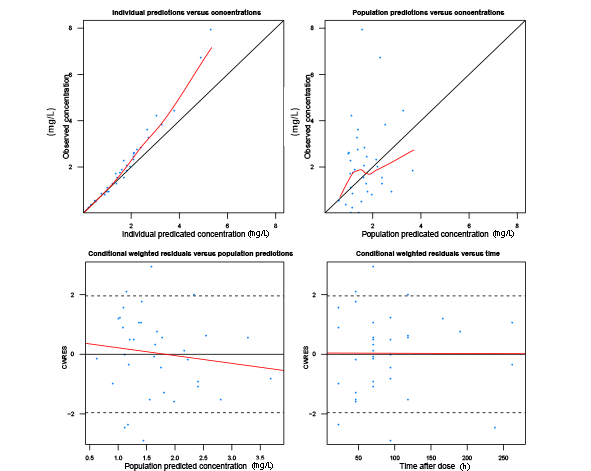

Supplement: Supplementary file 3 [file Image1.TIF]
